# Bilateral Variation in the Origin of Circumflex Femoral Arteries: Anatomical Insights and Clinical Implications

**DOI:** 10.7759/cureus.84178

**Published:** 2025-05-15

**Authors:** Amit K Pal, Anasuya Ghosh

**Affiliations:** 1 Anatomy, All India Institute of Medical Sciences, Kalyani, Kalyani, IND

**Keywords:** circumflex femoral artery, clinical implication, femoral artery, profunda femoris artery, variation

## Abstract

The medial circumflex femoral artery (MCFA) and lateral circumflex femoral artery (LCFA) are crucial in providing blood supply to the hip and femoral head region and the adductor and extensor compartments of the thigh. This case report aims to document a rare bilateral variation in the origin of MCFA and LCFA observed during a routine cadaveric dissection in an anatomy lab. In a male donor aged approximately 78 years, the MCFA on the left side originated from the profunda femoris artery (PFA), whereas the LCFA arose from the femoral artery (FA). On the right side, the MCFA originated from the FA, while the LCFA had a typical origin from the PFA. These arterial variations were measured with reference to standard anatomical landmarks. Awareness of such variations can help clinicians avoid complications during procedures involving the FA and PFA.

## Introduction

The profunda femoris artery (PFA), also known as the deep femoral artery, represents the largest branch emerging from the femoral artery (FA). It is critically involved in supplying blood to the muscles of the thigh, hip joint, and femoral head [1]. Typically, the PFA arises posterolaterally from the FA approximately 4-5 cm below the inguinal ligament. It subsequently gives rise to the medial circumflex femoral artery (MCFA) and the lateral circumflex femoral artery (LCFA) proximally within the thigh region. Together with its perforating branches, these arteries ensure the vascularization of the hip joint and thigh muscles involved in adduction, flexion, and extension [1,2]. While variations in the origin of the MCFA and LCFA are common, bilateral asymmetry in their origin patterns within the same cadaveric specimen appears to be lacking in the literature. Such variations become particularly significant in surgical contexts, including hip arthroplasty, catheter-based femoral interventions, and reconstructive surgeries [3]. Atypical origins of MCFA or LCFA may reduce blood flow to the femoral head, heightening the risk of avascular necrosis following hip surgery. Similarly, variant branching patterns of the PFA and its associated circumflex arteries can impact the success of vascular procedures like stenting or embolectomy [3,4]. This case report highlights a unique bilateral variation in the origins of the circumflex femoral arteries, stressing the clinical significance of detailed preoperative imaging to prevent complications in surgical settings.

## Case presentation

The anatomical dissection was conducted on a 78-year-old male donor in the anatomy laboratory at All India Institute of Medical Sciences (AIIMS), Kalyani, in Kalyani, India. The procedure involved the sequential and careful removal of the skin, superficial fascia, and fascia lata to delineate the triangle's boundaries bilaterally. The femoral sheath was carefully removed to identify the femoral vessels. The FA, PFA, and associated branches were meticulously dissected and preserved for examination. Using a digital caliper (Dijite stainless steel waterproof digital caliper, resolution 0.1 mm/0.01 inch, accuracy ±0.2mm), measurements were taken from the midpoint of the inguinal ligament to the origins of each relevant vessel under discussion (PFA, MCFA, and LCFA of both sides). Two independent observers carried out measurements to enhance accuracy and reduce observer bias.

Findings regarding the site of origin of the PFA, MCFA, and LCFA are summarized in Table 1, measured from the mid-inguinal point. The dissection revealed bilateral variations in the origins of the circumflex femoral arteries.

**Table 1:** The site of origin of PFA and circumflex femoral arteries MIL: midpoint of the inguinal ligament; FA: femoral artery; PFA: profunda femoris artery; MCFA: medial circumflex femoral artery; LCFA: lateral circumflex femoral artery

	Left	Distance of the left-sided artery origin from MIL	Right	Distance of the right-sided artery origin from MIL
PFA origin	Posteromedial aspect of the FA	28.5 mm	Posterolateral aspect of the FA	20.85 mm
MCFA origin	PFA	33.2 mm	FA	13.9 mm
LCFA origin	FA	11 mm	PFA	55.3 mm

**Figure 1:**
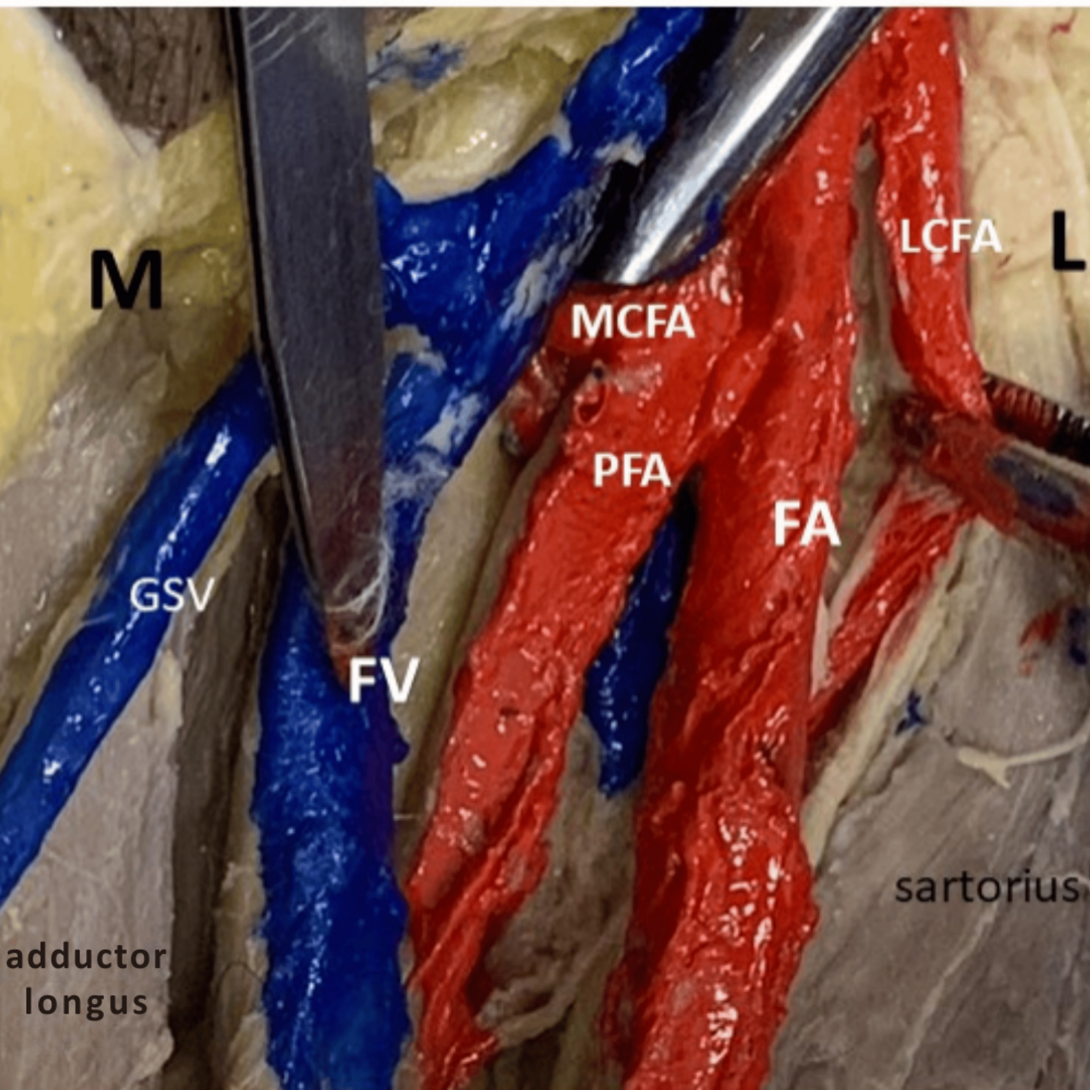
Origin of LCFA and MCFA on the left side M: medial side; L: lateral side; LCFA: lateral circumflex femoral artery; MCFA: medial circumflex femoral artery; PFA: profunda femoris artery; FA: femoral artery; FV: femoral vein; GSV: great saphenous vein

Figure 1 clearly demonstrates the origins of the LCFA and MCFA on the left side. The LCFA originates directly from the FA, while the MCFA arises from the PFA. The image also displays related structures, including the femoral vein (FV), great saphenous vein (GSV), and surrounding musculature, namely, sartorius and adductor longus.

**Figure 2:**
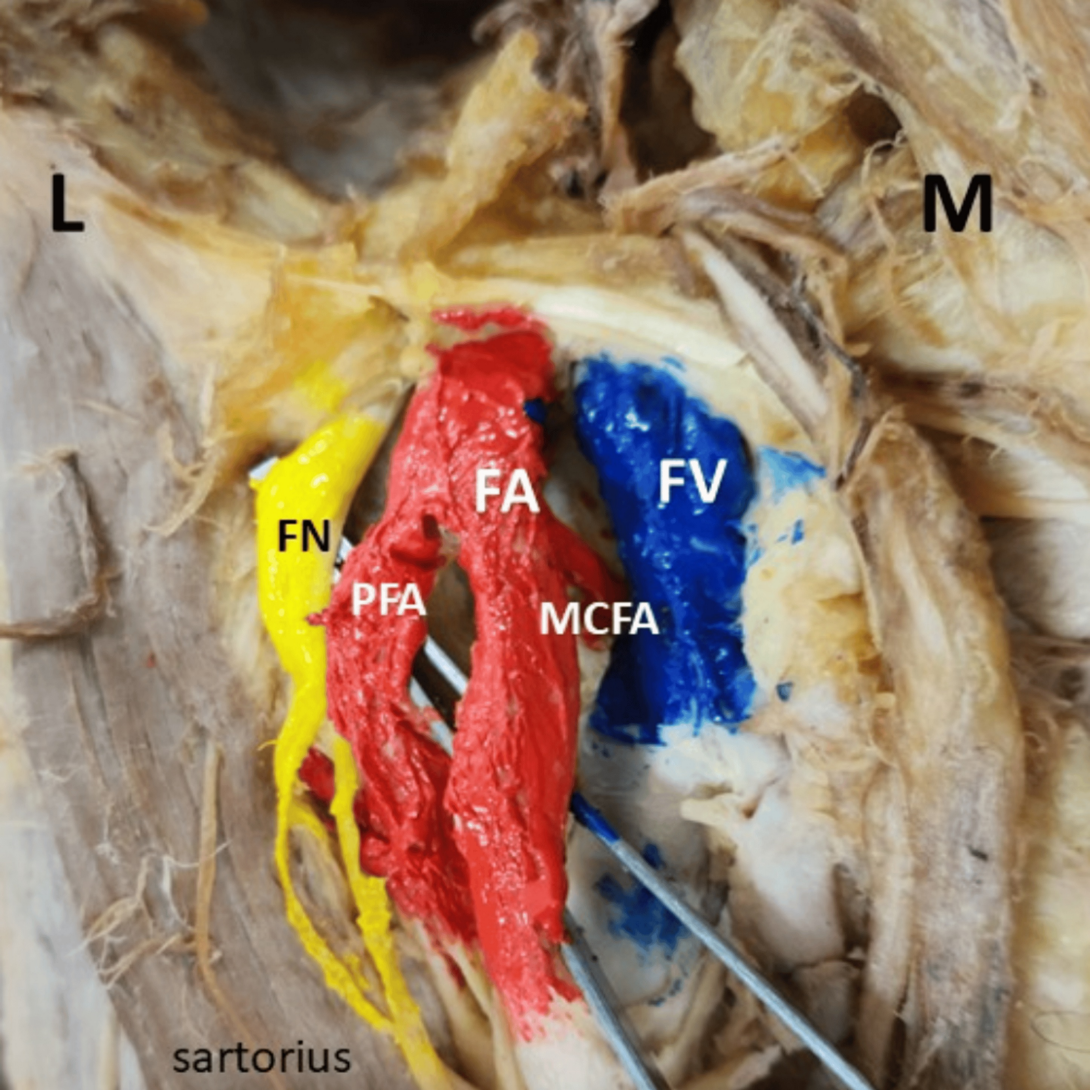
Origin of MCFA on the right side M: medial side; L: lateral side; MCFA: medial circumflex femoral artery; FA: femoral artery; FV: femoral vein; FN: femoral nerve; PFA: profunda femoris artery

Figure 2 shows the origin of the MCFA on the right side, originating from the FA. The FA is prominently displayed, along with the PFA branching off from it. The femoral nerve (FN) and FV are distinctly marked, respectively.

**Figure 3:**
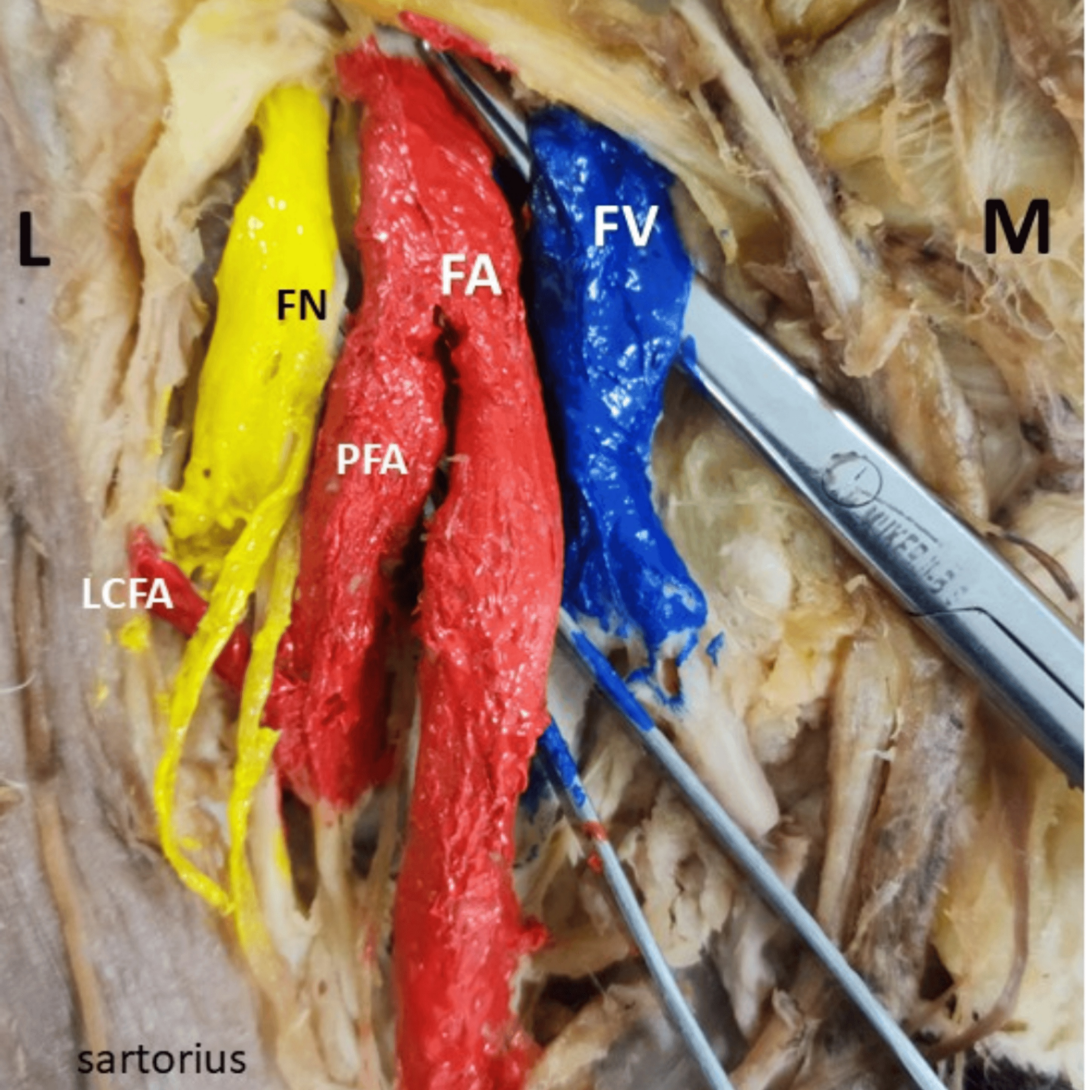
Origin of LCFA on the right side L: lateral side; M: medial side; LCFA: lateral circumflex femoral artery; FA: femoral artery; FV: femoral vein; FN: femoral nerve; PFA: profunda femoris artery

Figure 3 illustrates the origin of LCFA on the right side, arising directly from the PFA. FA, FN, and FV are clearly identified.

## Discussion

Variations in the origins of MCFA and LCFA have frequently been described and explained through embryological and developmental considerations [1,5]. During embryonic development, the vascular system of the lower limb originates from a primary axial artery derived from the dorsal aorta. This axial artery forms the initial vascular scaffold, branching into specific limb arteries [5]. The deep FA develops from the middle part of the embryonic axial artery, which undergoes medial rotation as the lower limb develops and rotates. Variations in the circumflex femoral artery origin are linked to the degree of failure of this middle axial part's medial rotation around the femoral axis [6]. In subsequent developmental stages, selective enlargement and regression within this primary capillary network are influenced by molecular signals, hemodynamic factors, and transcriptional pathways, leading to the final branching pattern of the vessel [5,6]. Based on their origin, circumflex femoral arteries have been classified into four principal categories (Table 2). Parameters such as the descending branch of the LCFA origin are excluded due to infrequent occurrence and limited clinical impact.

**Table 2:** Variations of the site of origin of the circumflex femoral artery LCFA: lateral circumflex femoral artery; MCFA: medial circumflex femoral artery; PFA: profunda femoris artery; FA: femoral artery This table has been created by the authors using information from [7].

Type	Description
Type I	LCFA/MCFA originates directly from the PFA
Type Ia	The MCFA arose proximal to the LCFA
Type Ib	The LCFA arose proximal to the MCFA
Type Ic	Both circumflex arteries arose as a common trunk from the PFA
Type II	LCFA/MCFA originates from the FA
Type IIa	The MCFA arose from the FA and the LCFA from the PFA
Type IIb	The LCFA arose from the FA and the MCFA from the PFA
Type IIc	LCFA/MCFA arises from a common trunk with another circumflex artery
Type III	Both LCFA and MCFA arose from the FA
Type IV	LCFA/MCFA has an ectopic origin (e.g., directly from the external iliac artery or unusual branches)

The MCFA most commonly originates from the PFA, as observed across various studies. However, it can also arise from FA, proximal or distal to PFA, though less frequently. Al-Talalwah reported that the MCFA originated from the PFA in 57% of cases and directly from the FA in 39.3% of cases. Less frequently, it originated from the superficial femoral artery (SFA) in 2.5% or the LCFA in 0.6% of cases. It was found to be congenitally absent in 0.6% [6,8]. Rare variations include the MCFA originating from the external iliac artery or sharing a trunk with the inferior epigastric artery, presenting significant surgical risk due to atypical positioning [9,10]. In our dissection, the left MCFA originated typically from PFA, 33.2 mm distal to the inguinal ligament. In contrast, the right MCFA originated unusually from FA at 13.9 mm below the ligament, posing potential risks during femoral surgical interventions.

Similarly, the LCFA typically arises from the PFA, though it can occasionally originate from the FA proximal or distal to the PFA. Rare instances of duplication have also been reported, complicating surgical dissection and increasing bleeding risks [4]. The origin of LCFA varies significantly from the midpoint of the inguinal ligament [8], necessitating thorough preoperative imaging for procedural safety [11]. Furthermore, given the proximity of LCFA to the FN, special attention is warranted during FN blocks or surgical exposure [12,13]. Both circumflex arteries can occasionally originate independently from the FA, with the profunda typically having a low origin. Bergman et al. noted no gender-related differences in the origins of the profound and circumflex arteries on either side [14]. In the present case, the LCFA exhibited variation by originating from the FA on the right side at 11 mm below the ligament and from the PFA on the left side, located 55.3 mm below the ligament, underscoring asymmetry and reinforcing the need for surgical caution.

Although the PFA typically originates posterolaterally from the FA within the femoral triangle, reported variations include origins from posterior, medial, lateral, anterolateral, or posteromedial aspects of the FA. Its origin relative to the inguinal ligament also varies widely [8], typically between 30 mm and 50 mm distally [15]. A bilateral differential origin was observed in our case, measuring 28.5 mm and 20.85 mm from the mid-inguinal point on the left and right sides, respectively. Such low origins of PFA influence the trajectory of its branches, complicating FA catheterization procedures. The PFA may originate as close as 12-15 mm below the inguinal ligament [16], running superficially parallel to the FA or beneath thigh muscles before piercing the adductor magnus [17]. Around 10% of cases exhibit a common trunk between PFA and MCFA, posing additional operative challenges [17]. Surgeons must remain vigilant about these variations to optimize outcomes.

Clinically, variations in MCFA and LCFA origins significantly impact orthopedic, vascular, and reconstructive surgeries and interventional radiology. Unrecognized anatomical deviations might compromise the blood supply to the femoral head, heightening the risk of avascular necrosis during hip arthroplasty [9]. Variations may complicate procedures like trochanteric osteotomies and acetabular fracture fixations or result in increased intraoperative bleeding [4]. Duplication of LCFA branches further complicates free-flap harvesting and reconstructive procedures due to unpredictable vessel courses, potentially causing ischemia [11]. Similarly, unrecognized arterial variations in interventional radiology can result in unintended tissue embolization or complications during vascular bypass grafting, leading to surgical failures [18].

Thus, comprehensive preoperative vascular imaging and thorough anatomical knowledge remain essential for avoiding complications and improving surgical precision.

## Conclusions

Recognizing arterial anatomical variations is crucial for clinicians to prevent surgical complications and achieve optimal outcomes, particularly in interventions involving the FA and PFA. This case report documents a rare bilateral asymmetry in the origin of the MCFA and LCFA, corresponding to type IIa and IIb variants, underscoring the importance of understanding cross-sided variability.

Such anomalies may affect procedures like femoral catheterization, flap harvesting, FN blocks, arterial bypass, and reconstructive surgeries. Variation in the MCFA origin can endanger femoral head perfusion, increasing the risk of avascular necrosis, particularly during embolization and hip surgeries. Radiologists, orthopedic surgeons, and vascular specialists must be aware of these deviations to prevent iatrogenic injury. This report highlights the necessity of comprehensive preoperative vascular imaging, anatomical classification of variant patterns, and integration of variant knowledge into procedural planning.

Future multicentric cadaveric and radiological studies should explore the population-specific prevalence and functional significance of such variations, with emphasis on their embryological basis and clinical ramifications.
